# Intersectoral collaboration and health system resilience during COVID-19: learnings from Ahmedabad, India

**DOI:** 10.1093/heapol/czae045

**Published:** 2024-11-18

**Authors:** Sandul Yasobant, K Shruti Lekha, Hardi Thacker, Bhavin Solanki, Walter Bruchhausen, Deepak Saxena

**Affiliations:** Department of Public Health Science, Indian Institute of Public Health Gandhinagar (IIPHG), Opp. Air Force Head Quarters, Nr. Lekawada, Gandhinagar, Gujarat 382042, India; Centre for One Health Education, Research & Development (COHERD), Indian Institute of Public Health Gandhinagar (IIPHG), Opp. Air Force Head Quarters, Nr. Lekawada, Gandhinagar, Gujarat 382042, India; School of Epidemiology & Public Health, Datta Meghe Institute of Medical Sciences (DMIMS), Meghe, Sawangi, Wardha, Maharashtra 442107, India; Global Health, Institute for Hygiene & Public Health, University Hospital Bonn, Venusberg-Campus 1, Bonn 53127, Germany; Department of Public Health Science, Indian Institute of Public Health Gandhinagar (IIPHG), Opp. Air Force Head Quarters, Nr. Lekawada, Gandhinagar, Gujarat 382042, India; Department of Public Health Science, Indian Institute of Public Health Gandhinagar (IIPHG), Opp. Air Force Head Quarters, Nr. Lekawada, Gandhinagar, Gujarat 382042, India; Department of Health, Ahmedabad Municipal Corporation (AMC), Danapith Rd, Old City, Danapith, Ahmedabad, Gujarat 380001, India; Global Health, Institute for Hygiene & Public Health, University Hospital Bonn, Venusberg-Campus 1, Bonn 53127, Germany; Department of Public Health Science, Indian Institute of Public Health Gandhinagar (IIPHG), Opp. Air Force Head Quarters, Nr. Lekawada, Gandhinagar, Gujarat 382042, India; Centre for One Health Education, Research & Development (COHERD), Indian Institute of Public Health Gandhinagar (IIPHG), Opp. Air Force Head Quarters, Nr. Lekawada, Gandhinagar, Gujarat 382042, India; School of Epidemiology & Public Health, Datta Meghe Institute of Medical Sciences (DMIMS), Meghe, Sawangi, Wardha, Maharashtra 442107, India

**Keywords:** Intersectoral collaboration, health emergencies, health system resilience, pandemic, COVID-19, India

## Abstract

Health system resilience refers to the capacity of a health system to effectively anticipate, assimilate, adjust to and recuperate from unforeseen disruptions and pressures. Evidence indicates that low- and middle-income countries (LMICs) have a double burden of dealing with the existing shortage of health resources in managing both non-emergency care and emergency care during epidemics. Intersectoral collaboration plays a pivotal role in managing crises such as pandemics. The World Health Organization has emphasized that effective intersectoral collaboration is vital for uninterrupted essential services during a pandemic. The study aimed to look at the collaborations entered into at various levels for managing the COVID-19 pandemic, taking as an example the municipal corporation in Ahmedabad, India. We undertook a qualitative study that involved conducting 52 in-depth interviews with officials from the health department, and other departments at the Ahmedabad Municipal Corporation (AMC), including firefighting, estate, engineering and education, as well as private stakeholders. Many diverse observations were documented in this study as collaboration varied across the sectors. A lack of hospital beds and shortage of essential drugs and oxygen posed challenges for healthcare providers and provided an opportunity to collaborate with private stakeholders. Mandatory COVID testing and mobile units such as the Sanjivani van and Vadil ghar seva were examples of some of the initiatives taken by the AMC to manage the pandemic that were instigated and implemented with support from various departments such as education, engineering, tax, estate, animal husbandry and firefighting. Proper communication between public and private entities will result in unfragmented efforts to combat emergencies. Thus, a resilient health system is necessary as well as systematic intersectoral collaboration to ensure the uninterrupted delivery of essential health services during health emergencies.

Key Messages

- Intersectoral collaboration plays a pivotal role in managing ongoing crises, such as health resource shortages, or emergency crises like epidemics and pandemics in low- and middle-income countries.
- There is a lack of understanding of the level of intersectoral collaboration necessary to maintain and/or optimize health system resilience. Therefore, the current study attempted to document examples of intersectoral collaboration within a local health system during the COVID-19 pandemic as a guide for future emergencies.
- There was evidence of some form of intersectoral collaboration at different levels of the health system (both public and private).
- A structured and systematic intersectoral collaboration is instrumental in the uninterrupted delivery of health services during both emergencies and for non-urgent care, ensuring a resilient health system.

## Introduction

Health system resilience refers to the capacity of a health system to effectively anticipate, assimilate, adjust to and recuperate from unforeseen disruptions and pressures that pose a risk to its capacity to provide vital healthcare services (World Health Organization (WHO), 2023). This encompasses the capacity to provide a diverse array of healthcare services, spanning from preventative measures to the management of chronic ailments, the presence of a proficient and adequately backed workforce comprising of physicians, nurses and other healthcare practitioners, and the capability to monitor disease patterns, assess the standard of care, and make informed decisions regarding resource allocation (Fridell *et al*., 2020).

The frequent occurrence of emerging and re-emerging infectious diseases could threaten the stability of healthcare systems (Kahn, 2006). India has recently experienced outbreaks of eight organisms of emerging and re-emerging disease in various parts of the country, six of which were of zoonotic origin (Dikid *et al*., 2013). The outbreaks have placed strain on the health system, testing its ability to deal with emergencies (Roychoudhury *et al*., 2020). A disproportionate infectious disease burden mainly affects LMICs like India, leading to disruption of service delivery (Mills *et al*., 2006). The outbreak of any disease has implications at individual and system levels. The supply and demand for healthcare services are affected during outbreaks or emergencies resulting in decreased access to routine health care, changes in healthcare service delivery, including availability of medicines, patients’ fear of contracting the disease and increased need for emergency care (World Health Organization (WHO), 2021). The literature indicates that LMIC countries especially have a double burden of dealing with an existing shortage of human health resources and handling additional infectious cases and non-emergency care at the same time. Since there were already insufficient health workers per capita in some of the most affected countries like India, the supply of health services was significantly reduced during the disease outbreaks (Hassell *et al*., 2017). Thus, the impact of unusual service demands on a healthcare organization’s operations (e.g. treating even a few patients with Severe Acute Respiratory Syndrome (SARS) or multiple burn patients in a non-burn facility) adds to the existing burden of unmet health-care needs (Šehović, 2017; Toner *et al*., 2017). Management of this challenging situation is a daunting task.

Concerns about poor governance, lack of workforce due to a high infection rate among healthcare workers, and a shortage of essential supplies during the recent pandemic were some of the issues highlighted by countries such as Bangladesh (Toner *et al*., 2017). Managing human resources and other resources, including finances, technology and communication, and considering human, animal and environmental health is crucial to combat any emergency. Quick decision-making and delivering targets for a resilient health system are essential as part of the pandemic response (Kouadio *et al*., 2012).

Collaboration was a vital and integral element in the successful management of COVID-19 and was possible with effective intersectoral cooperation. In Colombia, for example, the collaboration between academic and private institutions was helpful in dealing with the crisis (Turner *et al*., 2022). Similarly, collaboration helped to limit the pandemic’s impact in many other countries such as the United States (Koehlmoos *et al*., 2022). Collaboration between various players in the health sector, as well as with other departments such as social services, is an essential part of of managing a crisis (Alderwick *et al*., 2021). The World Health Organization (WHO) applauded the actions by the western Indian state of Gujarat during the later part of the COVID-19 pandemic (Indian Institute of Management Ahmedabad *et al*., 2022). Few initiatives were appreciated worldwide, and initial efforts to curtail the spread of infection through robust testing and tracking of international travellers were criticized (Israelsen and Malji, 2021).

Keeping these shortcomings in mind, our aim was to understand the different levels of collaboration entered into at institutional and/or individual level, including the private sector, in the city of Ahmedabad in western India, during the COVID-19 pandemic.

## Methods

### Study type

This cross-sectional study was conducted during 2020–22. A qualitative research technique was used to document the challenges faced by the various individuals working at administrative and clinical levels at the Ahmedabad Municipal Corporation (AMC) and private practitioners.

### Study setting

The study was conducted in the urban setting of Ahmedabad. The urban part of Ahmedabad is managed by a corporate body called the Ahmedabad Municipal Corporation that is also responsible for managing the city’s health system. The body is headed by the Commissioner, and the Medical Officer of Health looks after urban health and is a prime player during pandemic management.

### Study samples and sampling

A total of 52 in-depth interviews were carried out among administrative officials from health departments, including the deputy health officers of selected zones, and officials from non-health departments, such as education, estate, security, firefighting, engineering, transport, and media personnel who had worked during the pandemic in the AMC. Medical officers from the Urban Health Centres (UHCs) under the AMC and private practitioners in the city were included in the study. Other clinical staff, such as veterinary doctors who were employed by the AMC and worked during the pandemic, were also interviewed for the study. All study participants were recruited through purposive sampling, looking at the significant contribution to pandemic management.

### Data collection

An in-depth guide was developed to interview the administrative officials, medical officers and veterinary doctors. The interviews were carried out by a team of researchers from the authors’ institute, with a focus on understanding the collaboration patterns, plan of action and prioritizing activities, roles and responsibilities, and motivation to work during the COVID-19 pandemic. Officials flagged by study participants as part of the COVID task force or those who were involved in the management of the pandemic under the AMC were also included in the study. Each interview was conducted face to face for about 30–40 min in the vernacular language, and was recorded using a voice recorder. Verbal probes, such as repeating the statements, were used to elicit more information on crucial points.

### Data analysis

Transcription and translation was carried out for all the recorded in-depth interviews. Thematic analysis was conducted, and the data were coded based on that. The data analysis involved various team members to ensure reliability, reproducibility and stability. A deductive approach was used to obtain the information regarding the roles and responsibilities and intersectoral collaborations emerging from the data based on WHO health system building blocks. MAXQDA 22nd version was used to manage and analyse the data.

## Results

The sample consisted of 18 administrative officers from the AMC, including officials from the health as well as other departments, 16 medical officers from UHC, 14 private practitioners and 5 veterinary doctors. The interviews were conducted among all the consenting participants until thematic redundancy was obtained. The professional experience of the participants has been documented. The median for administration staff is 18.5 years, and the range of years of experience is 5 to 24 years. Similarly, the median years of experience for medical officers is 10 years, and the range is 2 to 33 years, whereas the median years of experience for private practitioners is 9 years, and the range is 4 to 34 years. In the case of veterinary doctors the median years of experience is 11 years and the range is 8 to 15 years. The average experience of non-health administrative officials at the AMC is 17.3 years, and that of health department officials is 16 years.This information was collected at the outset in order to understand the depth of their interaction with the health system.

The information gathered through interviews was documented and analysed regarding the building blocks of the health system (World Health Organization (WHO), 2010). The recorded perception was documented, and when analysed, the components were grouped based on the themes of leadership and governance, service delivery, health system financing, technologies, and health information systems. The collaborations under each building block were analysed to explore the functioning of the health system and its resilience in Ahmedabad city during the pandemic.

### Leadership and governance

The COVID-19 pandemic offered great potential for the health and non-health sectors to collaborate better in managing the outbreaks. The second wave of COVID-19 had a high incidence and mortality rate, and health department staff were overburdened. Staff from non-health sectors (such as tax, engineering, real estate, education, fire safety, security, animal husbandry, media, transportation, solid waste and police) were deployed to support COVID-19-related activities.

*‘ “ZAZA HATH RADIYAMNA” hence all department must should work together*…*if other department says this is not my work and do not take up the team duties than health department alone cannot do anything…’*. —Non-health Administrative Official

*‘Estate department helped in the testing of the small street shops and vendors and sewage department looked after social distance, tax department tested large commercial building so if anyone tested positive, we closed those places for 14 days, even for vaccination different department helped…’*. —Health Administrative Official

*‘…civil and SVP hospital staff were facing difficulty in travelling so we have provided our Amdavad Municipal Transport Service* [AMTS] *buses day-night, we department staff deployed the bus drivers, conductors and the administrative officials of the department with corporation team in different zones who were quarantine at home and distributed food packets…’*.

*‘all buses used for COVID were available at free of charge…buses were around 5691 from March, 2020 to October, 2020’*. —Non-health Administrative Official

*‘dry fodder exported from the out state, it was difficult and challenging, animal husbandry wrote letter to the commissioner other states and then shared with other state, then we got dry fodder for three months…’*. —Non-health Administrative Official

When a complete lockdown was imposed police were very strict about transportation; during lockdown, the animal husbandry department ensured that people received a regular milk supply, and also that pastoralists did not face difficulties when transferring milk from the house to the dairy farm. They also ensured minimal movement and maximum availability of cattle feed.

Apart from the animal husbandry department, real estate, engineering and AMTS staff also helped and provided food items to home quarantine patients, homeless people and patients admitted to hospital. The AMC estate and tax department supplied necessities like medicines, cooked food and other items to people under home quarantine, and they encouraged people to have the COVID-19 vaccine as well as to recommend it to owners of commercial buildings.

‘*Our staff used to make food like Poha/Khichdi and distributed by those who worked with NGO’*. —Non-health Administrative Official

‘*We ensured that pastoralists do not face any difficulties while transfer the milk from house to dairy and for that we stay connected with local administrations throughout lockdown and pandemic’*. —Non-health Administrative Official

‘*Estate and tax dept were providing things to isolated people at home, and tax dept was helping to bring people for vaccination from commercial buildings and offices…’. —*Non-health Administrative Official

### Service delivery

Firefighting department staff were involved in the fire safety of newly set up temporary COVID-19 hospitals and oxygen management in more than 300 bedded COVID-19 centres. They were also responsible for managing a fire safety protocol and plan in these centres, and they worked fulltime on these two tasks: oxygen storage and fire safety in COVID-19 centres.

The solid waste department was involved in waste management of home quarantine individuals and houses with foreign travellers. They received a list of COVID-19-positive home quarantine patients from the health department daily; additional staff were deployed for garbage collection so that disease transmission could be carefully controlled. The department was also involved in regulating crowds in public places as well as checking that the public adhered to COVID-19 precautionary measures such as wearing masks and keeping social distance in places like malls or markets. The animal husbandry department converted their nitrogen plant into an oxygen plant, providing an oxygen supply to the health department. Teachers from the education and Integrated Child Development Services (ICDS) departments were involved in mapping and contact tracing. The police department was involved in limiting unnecessary public movement during lockdown and protecting health workers from community resistance, as well as providing assistance to treat COVID-19-positive individuals or transfer them to hospitals. The malaria cell under the health department was involved in the sanitization of neighbourhoods with positive cases, as well as sanitization of public places. The waste management department was involved in mapping cases and managing waste from houses where COVID-19-positive patients were isolated. All these activities were carried out under the Commissioner’s leadership following the national and state directives.

Many volunteers and NGOs worked during the crises to support private hospitals and help them function smoothly. Collaboration between organizations and their private contacts helped private hospitals to manage food for patients and staff in hospitals. Oxygen supply during the second wave was carried out by the AMC or personal contacts of the heads of private hospitals. Veterinary doctors mentioned that apart from support from suppliers of medicines and pet food, they did not have any other substantial collaborations during the pandemic. During the pandemic, the service delivery was not only to provide the healthcare services that the health department was continuing to provide but also to manage the burden of patients due to the pandemic. All other non-health sectors were crucial in maintaining the supply chain for sustaining livelihood, which truly demonstrated the importance of intersectoral collaboration. This is the ultimate essence of maintaining health system resilience during a crisis, indicating that all health system players should collaborate in order to maintain the system tension.

### Health system financing

The non-health sectors helped the health sector by providing funds to manage the additional burden of the pandemic. They transformed their funds into COVID-related activities. Some of the local context documented in the study was: the estate and engineering departments temporarily paused luxury construction projects, and the animal husbandry department redirected resources to support campaigns, field workers’ dressing materials, and turning animal sheds into sanitizer installations. A deputy health officer also mentioned concerns about funding for health insurance, which needs to be significantly increased in poor communities.

‘…*regular events funds were converted into Covid-related work as events such as Kakariya Carnival, flower shop was completely closed’..” —*Health Administrative Official

*‘Major party-plot and community development work is part of the luxury, so we have never held basic amenities…’. —*Non-health Administrative Official

### Human resources

Human resources remained an issue for both private and public sectors during the pandemic. One crisis management strategy, among others, was to utilize senior medical and health residents in different phases of crisis management. HR agencies were supportive by providing human resources to private hospitals.

‘*There was labour shortage so the interns of the medical college were deployed in the management of COVID-19…’. —*Health Administrative Official

‘*There was multiple roles, even CEO, director and owner doctor of the hospital themselves had to change oxygen also so we did multiple roles with the same ultimate goal of saving patient’s life’.—*A Private Practioner

### Health information system

The media and healthcare system’s Information Education & Communication (IEC) cell worked round the clock to ensure that the correct risk communication reached the target community. They were involved in providing first-hand information regarding the burden of the disease to the public. Many innovative technologies were used to support collaboration across the sectors.

**Figure 1.**
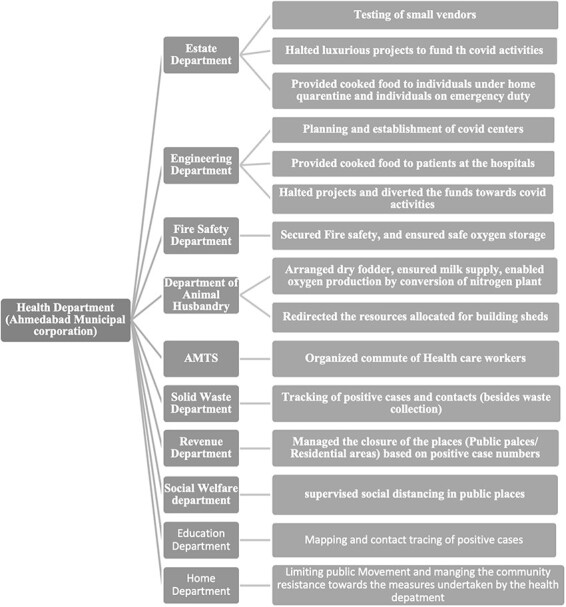
Intersectoral collaborations and activities undertaken by non-health sectors as an integral component of health system resilience during pandemic management in Ahmedabad city, India

Figure 1 shows an example of collaborative effort and activities of non-health sectors during management of the pandemic in Ahmedabad city. This indicates the support system that was established through intersectoral collaborations to maintain the health system’s resilience.

‘*We had a WhatsApp group, so it was all our colleagues, and partners from AMC so if there was any update, we would discuss it during lunch time’.—*A Private Practitioner

‘*WhatsApp groups and video calling was used to communicate with different stakeholders. The coordination was done based on the day to day burden’.—*A Non-health Administrative Official

## Discussion

This study documented the various collaborations at institutional and individual levels that were the key to pandemic crisis management and maintaining the health system resilience, which fulfils the WHO’s core advisory on multisectoral collaboration for health emergency preparedness (World Health Organization (WHO), 2020). Official documents produced in India prove that there was collaboration between the home department and police officials who were designated to work as the front line in all the states of the country. Yet there is not much evidence on collaboration with other departments (Public Health Foundation of India, 2020). A planned multi-sectoral approach yielded positive results in countries like Ethiopia (Ali *et al*., 2022). This study adds value to the importance of intersectoral collaborations in a local health system during the pandemic.

### Leadership and governance

**Figure 2.**
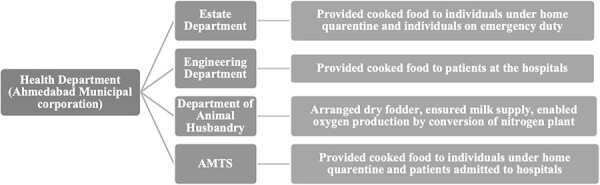
Intersectoral collaborations for managing the uninterrupted supply of food and other necessities during the pandemic to maintain the health system resilience of Ahmedabad, India

The various factors like provision of food, milk supply and other necessities are crucial during the pandemic like COVID-19. A report by a US clinical pharmacist and a report titled ‘The pandemic and the supply chain’ by the Johns Hopkins Bloomberg School of Public Health both mention the severe impact of the pandemic on the drug supply chain. The pharmacist’s report stated that a surge in the demand and limited supply of essential drugs, mainly those used in treating cardiovascular diseases, led to a dearth in the market (Bookwalter, 2021). This was similar to the information gathered during our study (Miller *et al*., 2020). In contrast, the animal health sector supply chain in the AMC was a success story where proper communication and cooperation resulted in just a minor delay in delivery and no shortage of animal food or medicines. Similarly, the collaboration between AMTS, the estate and engineering departments ensured an uninterrupted supply of food to patients in hospitals, patients and contacts on home quarantine, and staff working round the clock to meet the challenge. The intersectoral collaborations undertaken to manage the uninterupted supply of food are presented in Figure 2.

### Service delivery

The COVID-19 pandemic has presented many challenges for administrators. The dual burden of managing essential healthcare services and providing healthcare for COVID-19 infections was a daunting task. There were many hindrances to the smooth functioning of the healthcare delivery system; insufficient work force, lack of other resources, and especially a shortage of medicines and oxygen were experienced in many countries (Hossain *et al*., 2022). There was also a shortage of ICU beds and oxygen in many countries and a high mortality rate in other Indian states such as Punjab as well as in countries such as South Africa (The Economic Times, 2021; Rajatanavin *et al*., 2021). The scarcity of oxygen led to many deaths worldwide during the COVID-19 pandemic (AP News, 2020). The animal husbandry department was instrumental in producing more oxygen by converting a nitrogen plant. Similarly, support from the engineering department contributed to the establishment of COVID centres in Ahmedabad. These collaborations helped manage scarcity during the crisis.

Healthcare workers mapped positive cases, as well as tracked travellers in many Indian states as was the case in most other countries, e.g. Iran (Sabetian *et al*., 2021). Mandatory COVID testing prior to departure was introduced in many countries, including the United Kingdom, with inter-departmental support (Smith *et al*., 2021). Most routine services were disrupted during the first wave of COVID-19 and maintaining essential services was as challenging for other countries as it was for the AMC (Webb *et al*., 2022). Many countries tried to overcome the challenge by seeking support outside their health departments. Similar to the AMC and many other countries, Nepal suspended its immunization services during the first wave (Khatiwada *et al*., 2021; Ali *et al*., 2022; Public Foundation of India, 2022). The support of the solid waste, education, estate and revenue departments was helpful in tracking and tracing cases and contacts, enhancing testing of suspected cases and supervised social distancing in public places. All of this helped in managing the pandemic (Press Information Bureau, 2020; The Hindu, 2020; Times of India, 2022; Wallace *et al*., 2022). A study from Bangladesh reported that social stigma posed a challenge for healthcare workers (Sabetian *et al*., 2021). A reluctance to follow COVID preventive measures was also reported in studies from Iran and Ethiopia (World Health Organization (WHO), 2020; Ali *et al*., 2022). Other challenges such as patients not seeking care for fear of contracting the infection were reported in studies from Bangladesh and the United Kingdom. Resistance and reluctance to seek care was managed in Ahmedabad with the help of police personnel from the home department.

A report by the Food and Agriculture Organization documented the impact of COVID-19 on the delivery of veterinary services and animal diseases reporting (Miller *et al*., 2020). The report highlighted the negative impact on animal health services delivery due to movement restrictions, which was also reported by participants in our study (ReliefWeb, 2021). However, the human health sector was enriched due to the enormous support received from animal husbandry and/or other departments, as documented in this study. One of the collaborations observed in this study in the human health sector has huge potential for adoption by the veterinary sector during such a pandemic in order to maintain animal health services. Various agreements with private hospitals for managing the pandemic were similar to those carried out by Nepal’s emergency response as well as initiatives adopted by other Indian states such as Rajasthan, Punjab and Odisha (Press Information Bureau, 2020; The Hindu, 2020; Times of India, 2022; Wallace *et al*., 2022). Public Private Partnerships (PPP) add value to maintaining health system resilience and are well established in routine healthcare delivery and there might be potential to continue these during pandemic management (International Monetary Fund, 2021; World Bank Blogs, 2020).

**Figure 3.**
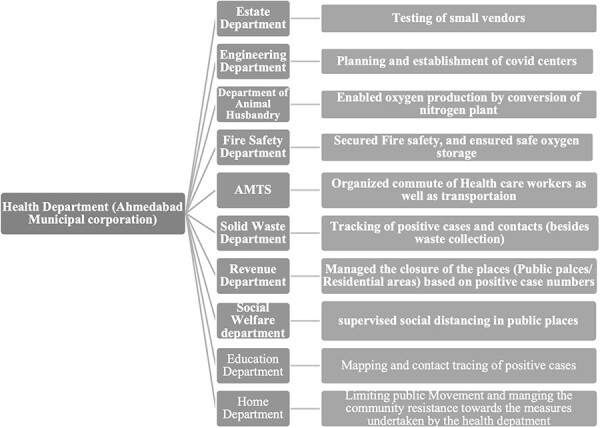
Intersectoral collaborations for uninterrupted service delivery during the pandemic to maintain health system resilience in Ahmedabad, India

Intersectoral collaborations for uninterrupted service delivery during the pandemic to maintain health system resilience in Ahmedabad, India are presented in Figure 3

### Health system financing

There was increased expenditure for private hospitals in Ahmedabad. The administration took care of the government hospitals’ financial flow by relocating their funds; the reallocation of funds by other departments like the estate and engineering departments was a relief. These departments halted luxury and non-essential construction and diverted their funds to COVID activities, as did the animal husbandry department thereby strengthening the finances needed to manage the pandemic.

The private hospitals faced issues in managing increased expenditure during the pandemic. The financial strain on private providers was documented in a report by the International Monetary Fund (IMF), (International Monetary Fund, 2021). The International Monetary Fund (IMF) report mentioned that fiscal authorities such as central banks implemented fiscal policies like increasing expenditure on healthcare and COVID management measures. The fiscal authorities in almost all countries were responsible for diverting funds towards COVID care (Centers for Disease Control and Prevention (CDC), 2019; US Office of Health Policy, 2022). This indicates the importance of financial sustainability in revamping collaborations during pandemic management.

**Figure 4.**
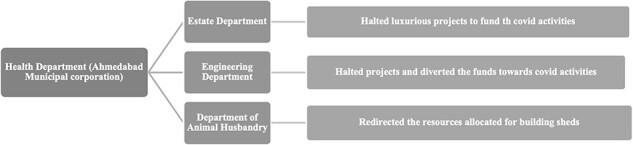
Collaborations for uninterrupted service delivery through financial assistance under AMC intersectoral collaborations to maintain health system resilience in Ahmedabad, India

Collaborations for uninterrupted service delivery through financial assistance under AMC intersectoral collaborations to maintain health system resilience in Ahmedabad, India are presented in Figure 4.

### Human resources

A shortage of staff in the health sector during the pandemic was experienced not only in Ahmedabad but also in high-income countries like the United States, as stated in the document released by the Assistant Secretary for Planning and Evaluation, US Office of Health Policy (US Office of Health Policy, 2022). The shortage of staff at the AMC was managed with the help and support of other departments such as education and estate. The estate department was involved in testing, and teachers from the education department were involved in contact tracing and line listing. Similarly, Anganwadi workers from the ICDS department were involved in the management of the pandemic. This support contributed to easing the HR crises in the public sector. The NGOs and HR agencies helped the private sector.

**Figure 5.**
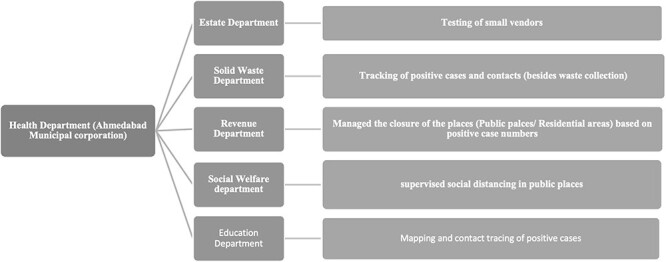
Collaborations for uninterrupted service delivery under the AMC through human resource allocation/assistance to maintain health system resilience of Ahmedabad, India

Collaborations for uninterrupted service delivery under the AMC through human resource allocation/assistance to maintain health system resilience of Ahmedabad, India are presented in Figure 5.

### Health information systems

Information systems were strengthened and various ways were adopted to provide health information. This was especially important in the animal health sector as effective health information was not provided at national or state level, unlike in the United States where the CDC issued guidelines for the management of pets during the pandemic. The veterinary doctors in Ahmedabad adopted the guidelines issued by the CDC (Escobar *et al*., 2021).

Teleconsultation for outpatient care enhanced multifold during the pandemic. Studies from Columbia and Singapore demonstrated how teleconsultation during the pandemic helped increase healthcare like in Ahmedabad(Ahmed et al., 2020; United Nations Development Programme, 2022). Here, the successful implementation of teleconsultation was possible with the help of the Telecom Regulatory Authority of India (TRAI), as in many other states (Dash et al., 2021)

The UNDP’s assessment of multisectoral collaborations in the COVID-19 pandemic response in selected countries reported that Bahrain, Saudi Arabia, UAE, Jordan, Lebanon, Tunisia, Sudan and Yemen also established inter-departmental committees to tackle the COVID crisis. The coordination mechanism was mandated by law in countries like the UAE and Jordan (United Nations Development Programme, 2022). Similarly, the AMC set up a multisectoral mechanism to manage the COVID crisis and also launched many novel initiatives. A proper sustainable communication and coordination system was not put in place, however. Appropriate multisectoral coordination will have a better impact on the health status of the community. Thus, the time-based collaborations carried out at the study site need to be sustainable in order to deal with an unprecedented crisis like the pandemic.

### Strengths and limitations of the study

The criteria for reporting qualitative research (COREQ) were followed for documenting the in-depth interviews. The interviews could not be restricted to specific topics, which helped produce diverse data. The study was restricted to the urban area of Ahmedabad, which is governed by the Ahmedabad Municipal Corporation. The results that emerged from the study indicate the importance of collaboration at individual and institutional levels for pandemic management. The hectic schedules of the officials and healthcare workers resulted in rescheduling interviews quite a few times. We acknowledge that there might be recall bias as data was collected between March and November 2022, but it also helped us to document more experiences of all the waves during the pandemic.

## Conclusion

Intersectoral collaboration remained a key aspect of healthcare delivery and management during the pandemic in maintaining the resilience of the health system. This study documented novel initiatives practised in an urban setting in western India as part of the collaboration strategies to meet the challenges faced during the pandemic. The city’s health department collaborated with many other non-health departments, but the collaborations were mostly need-based, according to the progress of the pandemic. Although collaboration ties are well documented in this study, the findings recommend how to sustain these collaborations in routine healthcare. Appropriate communication between public and private entities will result in unfragmented efforts to combat health emergencies as well as in routine health care. Thus, a resilient health system needs to be formed, and systematic intersectoral collaboration is necessary for the uninterrupted delivery of essential health services not only during health emergencies but also during routine health care to achieve optimal preparedness for upcoming health emergencies.

## Data Availability

The data used and/or analysed during the current study are available in an anonymized version from the corresponding author on request.

## Reflexivity statement

The authors include two women and four men with professional experience spanning multiple levels. While all authors specialize in various areas of public health, four specialize in health policy and systems research; one is a global health scientist, and another is closely involved with policy development and implementation in the public sector. This study has benefitted greatly from the deep expertise of the major authors from India within the field of public health practice, research and policy-making, including one author with great insight in global health.
